# The Associations of Auto-Brewery Syndrome and Diabetes Mellitus: A Literature Review and Clinical Perspective

**DOI:** 10.7759/cureus.41924

**Published:** 2023-07-15

**Authors:** Priyansh Patel, Diya Patel, Sai Dheeraj Gutlapalli, Ikpechukwu J Okorie, Arnold E Onana, Derek Ugwendum, Divine Besong Arrey Agbor, Foma Munoh Kenne, Philip Otterbeck, Jay Nfonoyim

**Affiliations:** 1 Department of Internal Medicine, California Institute of Behavioral Neurosciences & Psychology, Fairfield, USA; 2 Department of Internal Medicine, Baroda Medical College, Vadodara, IND; 3 Department of Internal Medicine, Gujarat Medical Education & Research Society, Sola, Ahmedabad, IND; 4 Department of Internal Medicine, Richmond University Medical Center, New York, USA; 5 Department of Pulmonary and Critical Care Medicine, Richmond University Medical Center, New York, USA

**Keywords:** auto-brewery syndrome, diabetes mellitus (dm), complications of diabetes mellitus, obesity and diabetes, candida species

## Abstract

Endogenous production of alcohol without the external intake of alcohol is called auto-brewery syndrome (ABS), and to get its levels to rise to a level that it has physical symptoms of alcohol intake is rare. The most common cause of ABS is the metabolism of ingested carbohydrates by intestinal microflora. This occurrence does not happen in all normal individuals but only in some high-risk individuals. Patients with diabetes mellitus (DM) have been hypothesized to be at high risk for ABS. We searched databases, such as PubMed, Medline, and PubMed Central, to search for existing literature with relevant keywords. In the finalized review, we have included 30 relevant articles. Alcohol formed in the gut gets absorbed in the bloodstream and immediately gets metabolized, so usually it does not achieve a level in blood high enough to cause symptoms. In high-risk patients, there is an increase in the level of bloodstream alcohol above a certain level, so it shows symptoms. Because there is higher blood glucose in DM, the patients have been shown to be at increased risk for developing ABS. Similarly, obesity is also a risk factor for DM, making it a high-risk condition for ABS. The most involved pathogens are *Candida* and *Saccharomyces*.

## Introduction and background

Auto-brewery syndrome (ABS) is a relatively rare medical condition that often presents a diagnostic, therapeutic, and medicolegal challenge [1,2]. Ethanol is a by-product formed as a result of the fermentation of certain metabolites. Similarly, in vivo, ethanol is also produced in the cells of the body via different metabolic pathways [3,4]. The condition in which ethanol produced in the body increases to a noticeable level without external consumption of alcohol is known as ABS [5]. The most common cause of ABS is intestinal microflora; the most common pathogenic species attributed are *Candida* and *Saccharomyces* [6]. As these pathogens are commensal flora and act by causing the fermentation of different dietary metabolites, this condition is also known as gut-fermentation syndrome [5,7]. It was first shown by Chapman et al. in the urine of racehorses. After racing, there was significant glycosuria, and upon analyzing the urine sample, a high concentration of alcohol was found when there was a delay in the sample analysis. These results showed that alcohol was produced through the fermentation of glucose that was present in the urine [8,9]. The most common metabolite that is fermented is carbohydrates [7]. Diabetes mellitus (DM) is a metabolic condition with elevated blood glucose levels [10]. As glucose is a favorable substrate for these pathogens, it has been postulated that individuals with DM have the tendency to produce detectable amounts of alcohol, despite not drinking, owing to ABS [2]. In Japanese literature, the condition of auto-fermenting is known as Meitei-sho. They documented two patients showing signs of alcohol intoxication, such as staggering gait, slurred speech, gastrointestinal (GI) distress, and state of confusion without ingesting alcohol, as one of the first cases of ABS in around 1972 [5,11,12]. In a normal person, the ethanol produced by commensal flora is absorbed and metabolized in the liver with the help of various enzymes, such as alcohol dehydrogenase (ADH), microsomal ethanol oxidizing system (MEOS), and hepatic catalase [6]. However, in individuals suffering from conditions where the metabolization of ethanol is reduced (undergoing long-term antibiotics, liver failure, and genetic deficiencies of liver enzymes) or conditions where the production of ethanol is increased (DM, obesity, short bowel syndrome, and Crohn’s disease), the development of ABS is found to be more common [6,7]. Our article primarily aims to establish a clinical perspective on the occurrence of ABS in patients with DM.

Methodology

Databases, namely, PubMed, Medline, and PubMed Central, were searched for the literature with keywords, including "Autobrewery Syndrome AND Diabetes mellitus," "Gut-fermentation syndrome AND Diabetes mellitus," "Autobrewery syndrome complications," "Autobrewery syndrome," "Complications of diabetes mellitus," and "Diabetes mellitus." First, duplicates were removed. After careful screening based on titles, abstracts, and inclusion and exclusion keywords, a total of 30 studies were included in the finalized review.

## Review

ABS occurs as a result of the production of endogenous (in vivo) ethanol by various metabolic processes, which include different disorders leading to the production of a precursor of ethanol and acetaldehyde [3,13]. Physiological-blood ethanol levels refer to the concentration of alcohol in the body of a normal person; this is because ethanol is constantly and spontaneously formed from acetaldehyde even in healthy subjects [3,14]. Sprung et al. suggested the physiological blood alcohol level to be 0.75 mg/L, while Shishkin et al., with the use of a more sensitive technique, found the range of physiological blood alcohol to be 0.08-1.30 mg/L [3,15]. Alcohol in healthy individuals gets metabolized by commensal flora and in the liver by hepatic enzymes [16-18]. Yajima et al. experimented and showed the production of ethanol in a sample containing glucose and candida; they observed that the ethanol concentration decreased after it peaked, suggesting that ethanol itself was being metabolized by candida pathogen [19]. ABS results when the blood alcohol level exceeds its physiological limits and when the production rate of alcohol exceeds the rate of metabolism of alcohol [3]. The rate of clearance of alcohol by the liver is shown to be 0.1 g ethanol per kg body weight per hour translating to about 7 g/kg/h for an adult individual of 70 kg [4,18]. DM is known to be associated with mainly microvascular, macrovascular, and neuropathic complications, including neuropathy, nephropathy, and retinopathy, along with atherosclerotic cardiovascular disease (ASCVD). Around two-thirds of diabetic patients die from myocardial infarction or stroke [10]. Apart from these major complications, DM is also associated with a plethora of disruptions in gut microbiota [20]. ABS can be evaluated by measuring breath alcohol levels or blood alcohol levels following a glucose challenge test, stool culture, and culture from upper and lower colonoscopies positive for bacteria or fungi [1,12].

Production of alcohol in ABS

Janet et al. and O’neal et al. hypothesized that microorganisms can produce ethanol from a variety of substrates and could be responsible as a major etiological factor for ABS [21,22], with glucose being one of the best substrates [19]. The most common species that are found responsible for the production of ethanol are *Candida *(*C. albicans*, *C. tropicalis*, *C. glabarta*, and *C. krusei*) and *Saccharomyces* (*S. cerevisiae*). These are all commensal microorganisms, part of intestinal microflora [6]. Among the pathogens, *Candida *is said to be the most common species that converts glucose to ethanol, with a production rate of 1 mg/h ethanol per gram of intestinal content and thus contributing to intestinal fermentation [23]. As diabetics have significant levels of glucose in their blood, Jones et al. said that there is a possibility of conversion of glucose to alcohol [3,18]. The ethanol that is produced due to intestinal fermentation of glucose gets absorbed into the central bloodstream, and then it moves to the liver and gets metabolized. Now, if the amount of alcohol reabsorbed exceeds the aforementioned capacity of the liver to metabolize, ethanol starts getting accumulated in the bloodstream [3,24]. Theoretically, at most, two molecules of ethanol are produced for one molecule of glucose fermented [19]. On the contrary, Logan et al. suggested that it is not credible for auto-brewing to produce such high concentrations of ethanol that can overwhelm the clearance capacity of the liver of healthy individuals [4].

Causes of ABS

**Figure 1:**
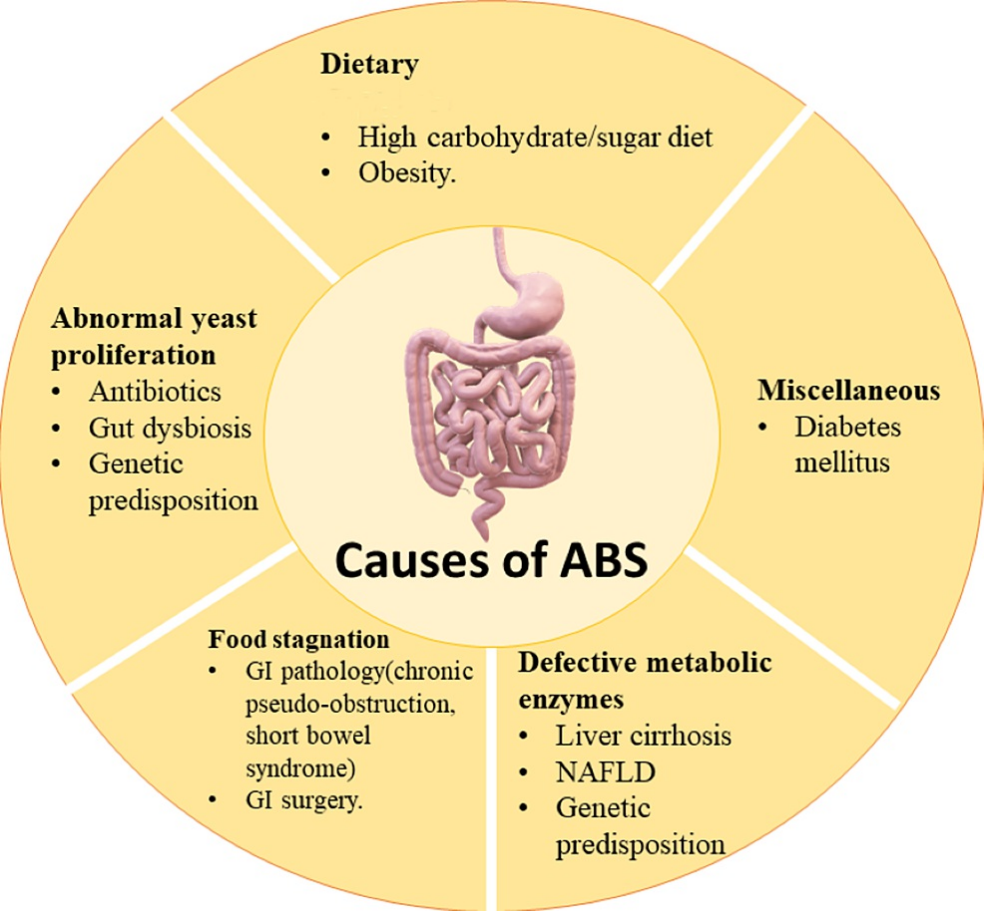
Causes of auto-brewery syndrome GI: gastrointestinal; NAFLD: non-alcoholic fatty liver disease Image credits: Priyansh Patel and Diya Patel

Kaji et al. said that the amount of ethanol that escapes the metabolism from the liver is too low to be detected by standard analytical methods [11]. It was found that in certain ethnic groups, such as Japanese, with genetic metabolic defect, elevated blood ethanol was found after ingestion of large amounts of rice [11]. Spinuci et al. and Dahshan et al. also showed similar findings in individuals with chronic pseudo-obstruction and young individuals with short bowel syndrome [25,26]. These individuals are at higher risk for development of ABS. The causes of ABS includes high carbohydrate/sugar diet, obesity, antibiotics, gut dysbiosis, genetic predisposition, GI pathology (chronic pseudo-obstruction and short bowel syndrome), GI surgery, liver cirrhosis, non-alcoholic fatty liver disease (NAFLD), genetic predisposition, and DM. Figure 1 shows the causes of ABS.

Symptoms of ABS

**Figure 2:**
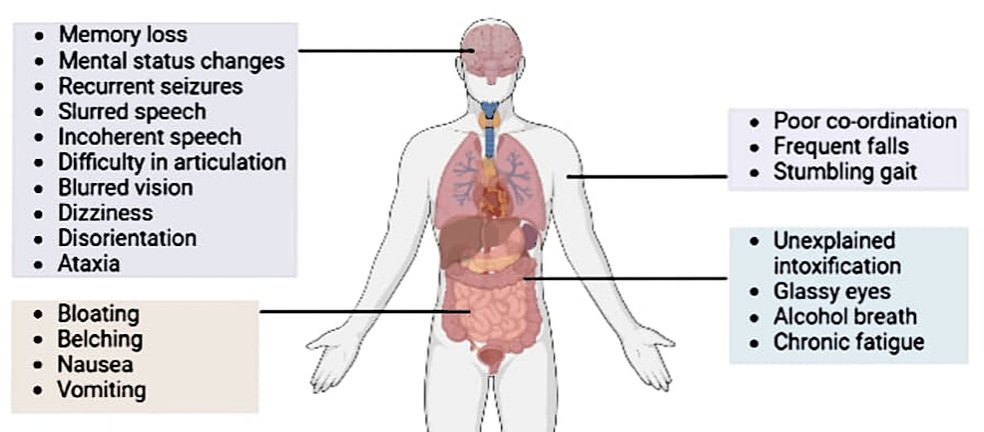
Symptoms of ABS Image credit: Priyansh Patel and Diya Patel (created using BioRender.com)

ABS can show signs of intoxication and withdrawal symptoms. It affects multiple organ systems, including the central nervous system (CNS), GI system, and musculoskeletal system, along with general symptoms [11,12]. Symptoms seen in patients of ABS are shown in Figure 2.

ABS in patients with DM

Even in the absence of alcohol consumption, endogenous ethanol may increase in diabetic patients [2,3]. The first observation of the association between ABS and DM was made by Alexander et al. in 1988. He observed the production of large quantities of alcohol in the urine samples of diabetic patients when stored at room temperature [9]. However, Yajima et al. said that no ethanol was found in samples with no glucose added to them [19]. The key microorganisms linked with DM are *C. albicans*, *S. cerevisiae*, *Lactobacillus fermentum*, *L. plantarum*, *L. casei*, *Roseburia intestinalis*, *Akkermansia muciniphila*, and *Bacteroides fragilis*. These bacteria have all shown the ability to effectively metabolize glucose and improve insulin sensitivity but might lead to the development of ABS by producing alcohol as a by-product in the process [20]. As one of the first-line treatments of DM, metformin is also known to cause alterations in the composition of gut microbiota through the modulation of inflammation, glucose homeostasis, gut permeability, and short-chain fatty acid-producing bacteria [20]. As diabetic patients are immunocompromised, they are highly susceptible to infections, such as invasive candidiasis, oral candidiasis, mucormycosis (*Rhinocerebral zygomycosis*), aspergillosis, oral lichen planus, delayed wound healing, periodontal disease, and gingivitis [27]. Out of these, *Candida* species is highly associated with patients with poor glycemic control. Kumar et al. showed that increased levels of glucose in saliva were associated with an increased risk of oral candidiasis infection [28,29]. In the past decade, the prevalence of DM has increased, thereby increasing the incidence of ABS, and the World Health Organization (WHO) predicts that DM will affect 10% of adults by 2030 [27,30]. Obesity is another risk factor for DM that causes insulin resistance, and hence ABS is said to be frequently seen in the setting of DM, obesity, and a high-carbohydrate diet [1]. Obesity can itself alter gut motility and sensitivity to certain neuropeptides that regulate intestinal motility, such as cholecystokinin and bombesin. There is an increased incidence of irritable bowel syndrome and colonic pseudo-obstruction in obese individuals, as shown by Satheesh et al. Hence, obese individuals are at an increased risk of intestinal bacterial overgrowth and thereby increased endogenous production of alcohol [7]. With the increase in body mass index (BMI), the ethanol concentration was found to increase; hence, obese diabetics were at a higher risk for developing ABS [7]. Furthermore, Yajima et al. showed that the production of ethanol was directly proportional to the initial concentration of glucose; that is, the more the initial glucose concentration, the more ethanol produced. Moreover, as the ethanol concentration increased, the glucose concentration decreased [19]. Kubiak-Tomaszewska et al. showed that in patients with a high risk of developing ABS (DM, obese, and GI pathology), blood alcohol levels were found to be significantly higher after ingestion of a carbohydrate-rich diet like rice [6]. Gut dysbiosis also causes ABS by altering the normal flora of the small intestine, bacterial overgrowth, and stagnation of food due to dysmotility of the intestines [12]. Satheesh et al. hypothesized that complications of DM, such as visceral neuropathy and dysmotility of the intestines, favor the increase in bacterial growth and hence cause fermentation of the ingested carbohydrates, resulting in the formation of alcohol [7]. Ahmed et al. showed that there were increased blood ethanol levels in diabetic patients despite never having consumed alcohol, and there was increased growth of *S. cerevisiae* in the fecal content and *C. intermedia*, *Klebsiella pneumoniae*, and *Enterococcus faecium* in the samples collected from gastric and intestinal endoscopies from diabetic patients [1]. Hafez et al. observed patients suffering from liver cirrhosis with and without DM and saw that there was significantly increased blood alcohol concentration in patients with liver cirrhosis with DM than without DM [3]. Thus, DM might add on to the severity of ABS on top of liver cirrhosis. Ahmed et al. showed that there was a remarkable improvement in patients with ABS with a no-carbohydrate diet and administration of antifungal agents, such as fluconazole and amphotericin B. Moreover, probiotics, high-protein meal, and fecal microbiota transplant are also proposed treatments of ABS, but more research needs to be done to these modalities [1]. This fact suggests that fungal agents, such as *C. albicans,* might be responsible in causing ABS in patients with DM, where there is an increase in the concentration of blood glucose [19]. Despite having statistically higher blood alcohol levels in patients with DM, Simic et al. suggested that these concentrations do not increase to an extent where it might affect the outcomes of legal proceedings. Apart from the legal aspect, ABS can also affect personal relationships, social interactions, and employment status [2].

Limitations

This study has some limitations, one of which is the absence of sufficient high-level evidence, such as randomized controlled trials or meta-analyses. All the identified studies were based on a limited number of observational studies, case reports, case series, systematic reviews, and animal studies available. These studies exhibited heterogeneity in sample size and the measurement of variables. Furthermore, not all the studies assessed had similar variables and secondary outcomes. This review only included papers written in all languages, but full text information could not be retrieved from articles other than the English language. In addition, more research is required to make an association of ABS with different types of DM. Only studies talking about ABS in relation to glucose lowering or diabetes were considered, and studies involving ABS in association to hepatic, renal, cardiac, and CNS pathologies were not considered.

## Conclusions

ABS is an unusual pathology of endogenous production of alcohol without any external intake of alcohol. It can be due to multiple etiologies, such as diet, abnormal yeast proliferation, unusual food stagnation in the GI tract, defective or absent metabolic enzymes that remove the physiologically produced alcohol, and miscellaneous diseases like DM that can cause ABS due to multiple etiologies. ABS has the potential to affect multiple organ systems, including the CNS, GI, musculoskeletal, hepatic, cardiac, renal, and endocrine systems. ABS in DM is mainly due to possible food stagnation in the GI tract due to defects in GI motility, higher incidence of DM in obese people, alterations in the microbiota of the gut, and increased susceptibility to *Candida* infections and increased glucose concentration in the blood. Other common pathogens that have increased susceptibility in DM, such as *Saccharomyces*, *Lactobacillus*, and *Bacteroides,* can potentially cause ABS but not as frequently as *C. albicans*. The association between DM and ABS is still inconclusive. We still need more larger and reliable observational studies with more homogenous population groups that are targeted to define the association between ABS and DM.
